# Increased Haematopoietic Supportive Function of USSC from Umbilical Cord Blood Compared to CB MSC and Possible Role of DLK-1

**DOI:** 10.1155/2013/985285

**Published:** 2013-04-03

**Authors:** Simone Maria Kluth, Teja Falk Radke, Gesine Kögler

**Affiliations:** Institute for Transplantation Diagnostics and Cell Therapeutics, Heinrich Heine University Medical Center, 40225 Duesseldorf, Germany

## Abstract

Multipotent stromal cells can be isolated from a variety of different tissues in the body. In contrast to stromal cells from the adult bone marrow (BM) or adipose tissue, cord blood (CB) multipotent stromal cells (MSC) are biologically younger. Since first being described by our group, delta like 1 homologue (DLK-1) was determined as a discriminating factor between the distinct cord blood-derived subpopulations: the unrestricted somatic stromal cells (USSC), which lack adipogenic differentiation capacity, and the BM MSC-like CB MSC. In this study, experiments assessing the haematopoiesis-supporting capacity and molecular biological analyses were conducted and clearly confirmed different properties. Compared to CB MSC, USSC lead to a higher expansion of haematopoietic cells and in addition express significantly higher levels of insulin-like growth factor binding protein 1 (*IGFBP1*), but lower levels of IGF2. The data presented here also indicate that DLK-1 might not be the sole factor responsible for the inhibition of adipogenic differentiation potential in USSC but nevertheless indicates a biological diversity among cord blood-derived stromal cells.

## 1. Introduction

Within the last two decades, cord blood (CB) has been well established as a valuable source of haematopoietic stem and progenitor cells applied in the treatment of blood-related diseases. Besides haematopoietic cells, CB also contains cells with stromal properties [1, 2]. While initially all these cells were termed unrestricted somatic stem cells, further research revealed significant differences between the cell lines, such as the competence for adipogenic differentiation and expression of the preadipocyte marker delta like 1 homologue (*DLK-1*) [3] and expression of the homeobox (*HOX*) genes [4]. 

As of today, CB-derived cells have been distinguished by these characteristics into unrestricted somatic stromal cells (USSC) and cord blood multipotent stromal cells (CB MSC). USSC do not respond to adipogenic induction and possess a *HOX*-negative expression pattern, while CB MSC form lipid vacuoles in adipogenic differentiation assays and express a variety of the 39 human *HOX*-genes, similar to bone marrow (BM) derived MSC. This classification of CB-derived stromal cells was also confirmed on a clonal level, therefore excluding an effect of heterogeneity of the bulk cultures [3]. Although multiple groups confirmed the generation of USSC and CB MSC from cord blood [5–7], the question whether the respective cells are already present in cord blood or if they are cell culture artifacts is still not finally answered and discussed controversially. For example, Karagianni et al. proposed that all native CB-derived cells are USSC rather than MSC-like cells and that they gain their ability for adipogenesis by extrinsic factors such as dexamethasone [8]. However, experiments confirming this hypothesis are missing.

The missing differentiation into adipocytes is the most obvious, yet functional, difference between USSC and CB MSC, and by current classification [9] excludes USSC from being regarded as MSC-like cell. Though, it has to be mentioned that the acronym MSC might be misleading. Originally introduced by Caplan [10] as abbreviation for “mesenchymal stem cells”, this term has been discussed controversially within the last years [11]. Although many cells of different origin are referred to as MSC characterized by similar properties like morphology, immunophenotype, and mandatory basic differentiation potential (chondrogenic, osteogenic, and adipogenic), subtle differences can be noted. In this context, a potential relation of different MSC-like cells has to be discussed; especially since evidence arose that USSC can be transformed into cells with full adipogenic differentiation capacity (manuscript in revision; Liedtke et al., 2013). Therefore, although less specific, MSC as abbreviation for “multipotent stromal cells” might be more appropriate for these cells, which in turn would include native USSC. 

The discrimination of USSC from CB MSC by the expression of *DLK-1* and the inverse correlation with the adipogenic differentiation capacity was the first evidence for two distinct stromal cell populations in cord blood [3]. The function of DLK-1 during adipogenic differentiation is well described [12–14]. Besides the inhibitory effect on adipogenesis, DLK-1 is known to play a major role during differentiation of hepatoblasts and considered a potent regulator of haematopoiesis [15–17]. 

The aim of this study was to further assess the function of DLK-1 and its soluble form, known as fetal antigen 1 (FA1) [18], in regard to a potential biological diversity between USSC and CB MSC which might have impact on future clinical use. Therefore, the haematopoiesis-supporting capacity of USSC and CB MSC *in vitro* was compared directly by co-culture experiments. Additionally, the expression of haematopoiesis relevant cytokines on molecular biological level was analyzed. 

## 2. Materials and Methods

### 2.1. Generation and Expansion of CB Stromal Cells

CB was collected from umbilical cord vein with informed consent of the mother. The bulk cell lines (USSC *n* = 61, CB MSC *n* = 29) used in this study were isolated from 90 different donors. USSC and CB MSC were generated by the same method as described previously [3], a classification into the two subtypes was only possible after examination of the adipogenic differentiation potential and HOX expression profile of each cell line. Briefly, mononuclear cells (MNC) of one cord blood were obtained by Ficoll gradient separation (density 1.077 g/cm^3^; Biochrom AG; Berlin, Germany) followed by lysis of remaining erythrocytes with ammonium chloride (pharmacy of the university medical centre Duesseldorf). 5–7∗10^6^ CB MNC/mL were cultured in DMEM low glucose (Lonza Inc.; Allendale, NJ, USA) with 30% FCS (Perbio/Fisher Scientific; Bonn, Germany), 1∗10^−7^ M dexamethasone (Sigma-Aldrich; St.Louis; MO, USA), penicillin/streptomycin and L-glutamine (PSG; all Lonza Inc.) until detection of adherent growing colonies. These cells were expanded without dexamethasone in a closed system applying cell stacks (Corning Inc.; Corning; NY, USA), incubated at 37°C in 5% CO_2_ in a humidified atmosphere and defined as one single, polyclonal bulk cell line. Reaching 80% confluence, cells were washed with phosphate buffered saline (PBS; Serag-Wiessner KG; Naila, Germany), detached with 0.25% trypsin (Lonza Inc.) and replated 1 : 3. 

### 2.2. Generation of Bone Marrow Stromal Cells

BM MSC lines were generated from bone marrow of healthy donors as described previously [1]. MNC were isolated and plated in DMEM low glucose with 30% FCS and PSG until detection of adherent growing colonies.

### 2.3. Biological Controls

Human skin fibroblasts (NHDF) were provided by the Department of Dermatology, Heinrich Heine University Duesseldorf. HepG2 and nTERA-2 cell lines were purchased from ATCC and cultured according to the manufacturer's instructions. 

### 2.4. Generation of Cell Clones Using the AVISO CellCelector

Clonal populations were obtained from established cell lines in early passages applying the AVISO CellCelector (ALS GmbH; Jena, Germany) as described previously [3]. Briefly, cells of one single bulk cell line were plated at low density (166 cells/cm^2^) in 6-well cell culture plates (Corning Inc.) and after allowing the cells to get adherent again, distinct single cells were selected, picked and transported to a defined destination well of a 96-well cell culture plate (Corning Inc.) and cultured with preconditioned medium. Pictures were taken before and after each picking process to document successful single cell selection. The term “USSC 1 clone 1” defines the first clonal population isolated from USSC cell line 1, this nomenclature also applies to CB MSC-derived clonal populations. All cell lines were used between passage 5 and 8. 

### 2.5. *In Vitro* Differentiation into Adipocytes

Differentiation into adipocytes was performed as described previously [2]. Adipogenic differentiation was induced applying DMEM high glucose (4.5 g/L, Lonza Inc.), 10% FCS, PSG, 1∗10^−6^ M dexamethasone, (water soluble, cell culture tested), 0.2 mM indomethacin, 0.1 mg/mL insulin, and 1 mM 3-Isobutyl-methylxanthine (all Sigma-Aldrich). Media was changed twice a week, alternating with cultivation media containing 0.01 mg/mL insulin. Lipid vacuoles were stained with Oil Red O (Sigma Aldrich) after 21 days. As negative control, the cells were cultured in DMEM low glucose (1 g/L, Lonza Inc.), 10% FCS, and PSG.

### 2.6. Co-Cultures of CD34-Selected Cells on Stromal Feeder Layers

Stromal feeders were prepared in 24-well plates by seeding 5∗10^4^ cells (USSC, CB MSC) in standard culture media. After 24–48 h, when density was app. 80–90%, a growth arrest was induced by irradiation (30 gy; cobalt source).

Cord blood-derived CD34^+^-cells were isolated from the fraction of the mononucleated cells following Ficoll-gradient centrifugation and erythrocyte lysis by ammonium chloride using a CD34 progenitor kit for magnetic-activated cell sorting (MACS) and Midi-MACS magnets with LS columns (all Miltenyi Biotec; Bergisch Gladbach, Germany), according to the manufacturer's instructions. Isolated CD34^+^-cells were seeded on the irradiated, adherent feeders at 1∗10^5^/3 mL/well in fresh media for each day examined. CD34^+^-cells and feeder cells were clearly distinguishable microscopically in regard to size and form. On days 3, 7, 10, and 14, non-adherent cells were harvested, enumerated, and assayed by flow cytometry as well as seeded in colony-forming unit assays.

### 2.7. RNA Isolation and Real Time PCR

Total RNA of cells was isolated utilizing the RNeasy Mini Kit (Qiagen; Hilden, Germany). 1 *µ*g RNA was reverse transcribed applying SuperScriptIII (Invitrogen/Life Technologies; Carlsbad, Ca, USA) according to the manual.

Real time PCR was performed with SYBR Green PCR Mastermix (Applied Biosystems/Life Technologies; Carlsbad, Ca, USA; primer see Table 1). Evaluation of Taq Man Gene Expression Assays (Applied Biosystems/Life Technologies) was performed utilizing the SDS2.3 program and expression normalized to GAPDH. As comparison total fetal liver RNA extract was obtained from Stratagene (Stratagene/Agilent Technologies; La Jolla, CA, USA).

### 2.8. Immunofluorescence

For immunofluorescence detection, 10,000 cells/cm^2^ were plated on chamber slides (Lab-Tek,Chamber Slide, 2 Well Glass Slide; Thermo Scientific; Waltham, MA, USA). Cells were fixed with 4% PFA for 15 minutes. Primary antibody (dilution 1 : 250; Table 2) incubation was performed at 4°C overnight. Secondary antibody (Table 2) was applied in 1 : 1000 dilutions. Nuclei were stained with ProLong Gold antifade reagent with DAPI (Invitrogen).

### 2.9. Flow Cytometric Analysis and Colony Forming Unit Assay

Cell count was performed applying a Neubauer chamber. 0.5–1.0∗10^5^ cells were resuspended in 100 *μ*L PBS and stained with CD45-FITC and CD34-PE antibodies (5 *μ*L each, both BD Biosciences; Franklin lake, NJ, USA). After incubation for 15 minutes at 4°C, samples were washed with PBS and after centrifugation analyzed for percentage of CD34^high^/CD45^low^ cells using a FACSCanto flow cytometer (BD Biosciences). Additionally, cells were seeded at adjusted concentration (ranging from 100/mL on day 0 to 10.000/mL on day 14) in semi-solid, growth-factor enriched methyl cellulose (Methocult GF H4434; STEMCELL Technologies Inc.; Vancouver, Canada) and after 14 days of incubation (37°C, 5% CO_2_, humidified air) scored for the formation of colonies as described before [1].

For detection of DLK-1-expression on stromal cells, 1∗10^5^ cells in 100 *μ*L PBS (Serag Wiessner) were incubated for 30 minutes with 1 *μ*L DLK-1 antibody (clone PF299-1; Enzo life sciences; Farmingdale, NY, USA) and washed with PBS. As secondary antibody, 1.5 *μ*L of FITC-conjugated goat-anti-mouse antibody (Jackson ImmunoResearch Inc; West groove, PA, USA) was applied and incubated for another 20 minutes, followed by washing with PBS and subsequent centrifugation. 

For intracytoplasmatic staining, cells were fixed and permeabilized utilizing the BD Cytofix/Cytoperm kit (BD Biosciences) according to the manufacturer's instructions prior to addition of antibodies. As fixative, 4% paraformaldehyde (PFA; Otto Fischar GmbH; Saarbruecken, Germany) was added after washing, removal of PBS, and resuspension, resulting in a final concentration of app. 2% PFA. 

Finally, flow cytometry analysis was performed on a FACSCanto flow cytometer (BD Biosciences) employing the FACSDIVA software (version 5.0.3) for recording and WinMDI 2.8 (Freeware; Joseph Trotter) for analysis. 

### 2.10. Culture with DLK-1 Enriched Supernatant

CB MSC overexpressing DLK-1 were cultured in standard expansion medium for 4 to 5 days until reaching 80% confluence. The supernatant was collected and filtered using a syringe filter (Thermo Scientific). Concentration of soluble DLK-1 was determined applying DLK-1 ELISA. 

CB MSC were plated on 6 well cell culture plates and cultivated for 24 hours to allow adherence. Subsequently, incubation with DLK-1 positive medium was carried out for 30 minutes, 4 hours, and 24 hours, respectively. The cells were washed twice with PBS and lysed using RIPA cell lyse buffer (Invitrogen/Life Technologies) for western blot analyses or cell lyse buffer (Qiagen) for PCR approaches. 

### 2.11. DLK-1 ELISA

To perform enzyme-linked immuno-sorbend assay (ELISA) analysis of the supernatants, CB MSC and USSC were cultured under standard conditions (Human Pref-1/DLK-1/FA1 Quantikine ELISA Kit; R&D Systems Inc.; Minneapolis, MN, USA). After 3 days, the supernatant was collected, filtered and stored at −80°C until further procedures. Subsequently, the manufacturer's instructions were followed. Maternal plasma was employed as positive control. 

### 2.12. Western Blot Analysis

Total protein was analyzed in Western Blot analysis (NuPAGE System, Invitrogen/Life Technologies). For detection ECL PLUS Western Blotting Detection Reagents (GE Healthcare; Buckinghamshire, UK) was applied in accordance with the manufacturer's instructions.

### 2.13. DLK-1 Overexpression

As described previously [3], full-length human DLK-1 was amplified using Phusion Taq Polymerase (New England Biolabs). Insert and vector (pCL7Egwo) were digested employing Fast Digest EcoRI and Fast Digest XhoI (both Fermentas) with the *eGFP* gene replaced by the *DLK-1* gene. Products were then electrophoresed on 2% agarose gels and purified using the QIAquick Gel Extraction Kit (QIAGEN). Insert and vector were ligated at a 3 : 1-ratio applying T4 DNA Ligase (New England Biolabs). Transformed electrocompetent Top10 bacteria (Invitrogen) were plated on LB agar containing ampicillin (500 *μ*g/L) overnight and DLK-1 positive colonies were expanded in 100 mL LB media. Vectors were isolated applying MaxiPrep Kit (QIAGEN) and 293T cells were transfected with pCL7Egwo_DLK-1, GalvTM and CD/NL-BH utilizing FuGene HD Transfection Reagent. CB MSC were then transfected with the resulting virus loaded supernatant.

### 2.14. Cell-Sorting for DLK-1-Expression by Fluorescent Activated Cell Sorting (FACS)

CB MSC overexpressing DLK-1 were labelled with antibodies according to the protocol described above and sorted at the Core Flow Cytometry Facility of the University Medical Center Duesseldorf applying a MoFlo XDP cell sorter (Beckman Coulter) with Summit Software (5.1.0).

### 2.15. Statistics

Statistics were evaluated with the Graph Pad PRISM software (version 5.01; GraphPad Software Inc.; La Jolla, CA, USA). A Student's *t*-test was used and all values given as mean ± SD if not stated otherwise.

## 3. Results

### 3.1. DLK-1 as Marker to Distinguish USSC from CB MSC

**Figure 1:**
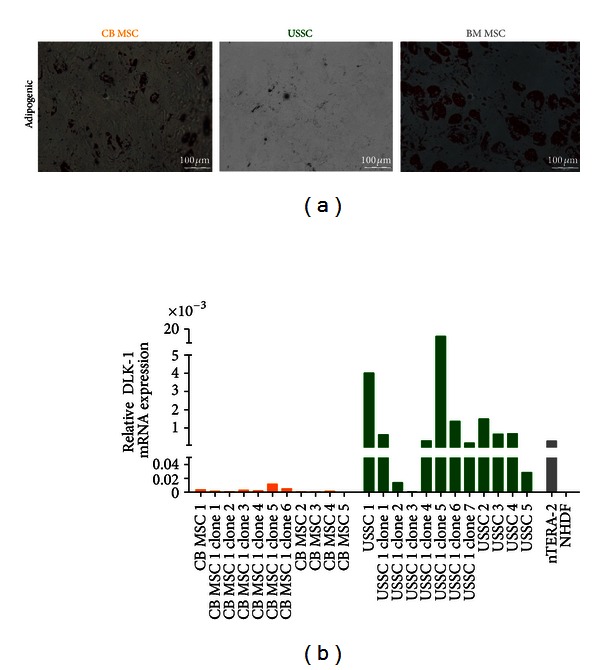
DLK-1 is a marker to distinguish USSC from CB MSC in cord blood. (a) Differentiation capacity of CB-derived stromal cells. Basal characterization of CB-derived stromal cells revealed the adipogenic differentiation as discriminating characteristic between CB MSC    (*n* = 29) and USSC (*n* = 61). CB MSC exhibited a high adipogenic differentiation potential, comparable to BM MSC, while USSC never formed lipid vacuoles following adipogenic induction as demonstrated by Oil Red O staining after 21 days of differentiation. (b) DLK-1 expression inversely correlated with the adipogenic differentiation potential. *DLK-1* mRNA expression was significantly higher in USSC and clonal USSC lines compared to CB MSC and corresponding clonal populations. The *DLK-1* expressing teratocarcinoma cell line nTERA-2 was tested as positive control, dermal fibroblasts (NHDF) served as biological negative control.

The presence of distinct stromal cell populations in cord blood (CB) has been described before [3, 4, 19]. Unrestricted somatic stromal cells (USSC) and CB multipotent stromal cells (MSC) share the same immunophenotype as well as osteogenic and chondrogenic differentiation potential *in vitro*. The two subpopulations can be classified by analyzing the adipogenic differentiation capacity and the *HOX*-gene expression profile. In contrast to CB MSC (*n* = 21) and MSC from bone marrow (BM MSC) (*n* = 44), USSC (*n* = 69) lack the adipogenic differentiation potential (Figure 1(a)) and as a general feature express high levels of *DLK-1 *on transcript level.

To evaluate the impact of heterogeneity of these subpopulations, USSC- (*n* = 29) and CB MSC-derived (*n* = 58) clonal populations were reanalyzed. USSC-derived clonal populations never exhibited the adipogenic differentiation potential, though a heterogeneous *DLK-1* expression was detected (Figure 1(b)), questioning the inhibitory function of DLK-1 in USSC. 

### 3.2. DLK-1 in USSC and Correlation with the Lack of Adipogenic Differentiation Capacity

DLK-1 is a transmembrane protein containing a soluble domain [20]. The cleavage of DLK-1 into a transmembrane and a soluble protein (termed PREF-1 in mice and FA1 in humans) is described as being mandatory to inhibit the adipogenic differentiation of stromal cells [20]. Overexpression or addition of DLK-1 in murine stromal cells results in an activation of ERK1/2, thereby downregulating the expression of adipogenesis-associated genes and inhibiting the adipogenic differentiation [21].

**Figure 2:**
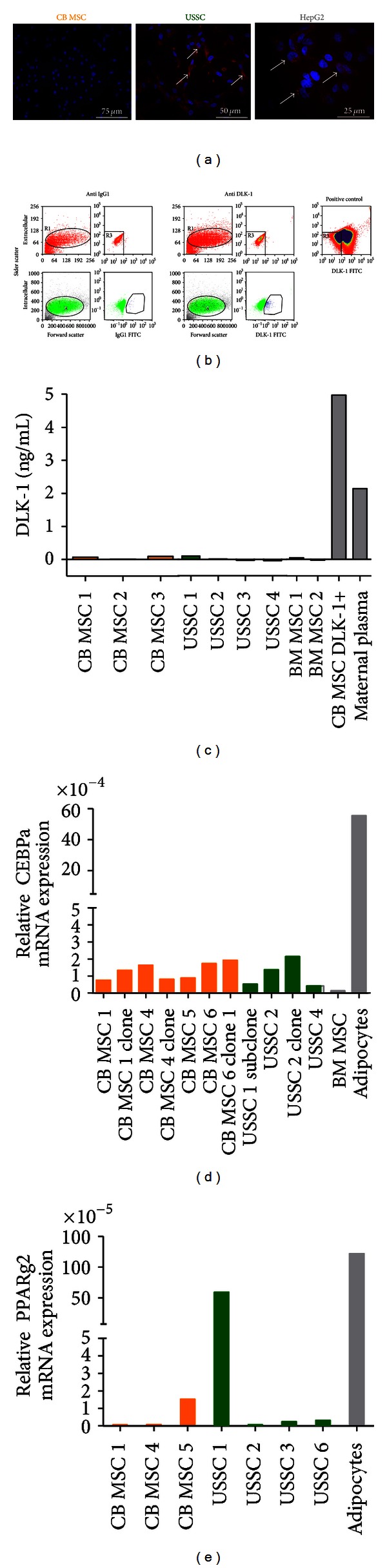
DLK-1 in USSC: inhibitor of adipogenesis? (a) Representative immunofluorescence staining of CB MSC (*n* = 3), USSC (*n* = 4) and HepG2. USSC revealed a clear DLK-1 expression, detected by immunofluorescence, while CB MSC were completely negative. The hepatocarcinoma cell line HepG2 served as biological positive control. (b) DLK-1 flowcytometry analysis was applied to clearly distinguish between intracellular and extracellular expression of DLK-1 protein. No DLK-1 expression could be determined in USSC by extracellular staining. After permeabilization a small DLK-1 positive subpopulation was detected in the USSC lines by intracellular staining. DLK-1 overexpressing CB MSC were used a positive control for extracellular staining. (c) By ectodomain shedding, the soluble and adipogenic inhibitory DLK-1 protein is cleaved. To validate whether USSC express the functional DLK-1, an ELISA was performed. The supernatant of 3 days standard cultures of USSC (*n* = 4) and CB MSC (*n* = 3) was analyzed, maternal plasma was used as positive control. No specific DLK-1 release was detected in any of the analyzed USSC or CB MSC, questioning function of DLK-1 expression in USSC. However, a clear secretion of DLK-1 was detected in the CB MSC overexpressing DLK-1. (d) A heterogeneous expression of *CEBP*α** (CB MSC *n* = 4, CB MSC-derived clones *n* = 3, USSC *n* = 2, USSC-derived clones *n* = 2) could be detected in different USSC and CB MSC cell lines analyzed by real time PCR analysis. (e) Real time PCR analysis revealed a heterogenous expression of *PPAR*γ*2* expression (CB MSC *n* = 3, USSC *n* = 4) in the USSC and CB MSC cell lines.

To analyze the DLK-1 protein level in USSC, immunofluorescence assays were performed. USSC (*n* = 4) exhibited a DLK-1 protein expression, while CB MSC (*n* = 3) were DLK-1 negative (Figure 2(a)) as described before [3]. In order to validate these data, the DLK-1 expression was additionally analyzed by flow cytometry applying a monoclonal antibody detecting the extracellular domain of DLK-1/FA1. Surprisingly, no extracellular expression of DLK-1 protein in USSC could be verified (Figure 2(b)), yet in some of the USSC lines examined, intracellular antibody labeling revealed a small but clearly DLK-1-positive subpopulation of USSC, suggesting intracellular localization of DLK-1/FA1. 

*In vivo*, DLK-1 is released into the circulation [18]. An ELISA-test detecting DLK-1/FA1 was applied to address the question whether the missing extracellular expression of DLK-1 in USSC might be ascribed to a high cleavage rate. However, secretion of DLK-1/FA1 was detected neither in supernatant of USSC cultures (0.014 ± 0.064 ng/mL), nor of CB MSC cultures (0.058 ± 0.042 ng/mL) (Figure 2(c)). Maternal plasma and supernatant of DLK-1-overexpressing cells served as positive controls with an approximately 40- and 80-fold higher expression, respectively (2.112 and 4,910; each *n* = 1).

In addition, the DLK-1 expression of USSC and CB MSC did not correlate with expression of the adipogenic transcription factors *CEBP*α** (CB MSC *n* = 4, CB MSC-derived clones *n* = 3, USSC *n* = 2, USSC-derived clones *n* = 2) and *PPAR*γ*2* (CB MSC *n* = 3, USSC *n* = 4). Here, the analysis of quantitative *CEBP*α** and *PPAR*γ*2* expression by real time PCR did not reveal any significant differences between the two subpopulations but instead demonstrated very heterogeneous expression levels already between the different cell lines analyzed (Figures 2(d) and 2(e)). 

**Figure 3:**
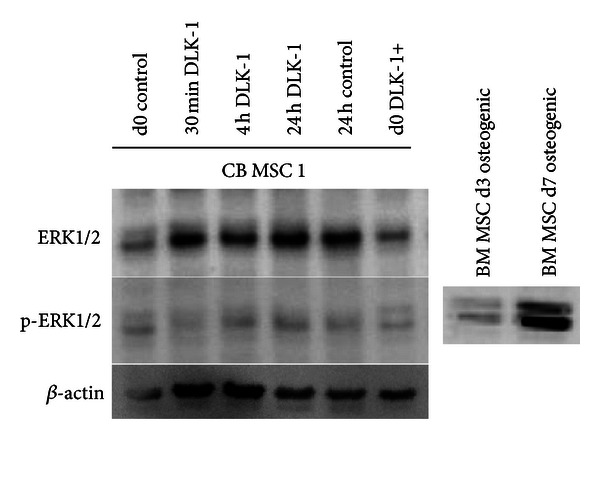
Regulations of DLK-1 in human and mice are not the same. To evaluate mechanisms induced by DLK-1 in human CB MSC upon exposure to DLK-1/Pref1, Western Blot analysis of ERK1/2 and p-ERK1/2 was performed (*n* = 3 experiments). CB MSC were treated with conditioned media of either the control cells or the DLK-1 overexpressing CB MSC. CB MSC (d0 control) and DLK-1 overexpressing CB MSC (d0 DLK+) were lysed and western blot analysis was performed applying full protein lysates after 30 minutes, 4 hours and 24 hours of incubation with conditioned media, respectively. ERK1/2 was already highly expressed in the non treated cells and remained expressed also in the cells treated. No DLK-1/Pref1 specific upregulation of p-ERK1/2 was detected in the CB MSC. As biological positive control, osteogenic differentiated (day 3 after induction) BM MSC were used. Internal loading control: *β*-actin.

To evaluate the phosphorylation status of ERK1/2, CB MSC were treated with cell culture supernatant of CB MSC overexpressing DLK-1 (with extracellular expression of DLK-1 and secreting soluble functional DLK-1 as confirmed by ELISA; Figures 2(b) and 2(c) and ERK1/2 expression was analyzed. For western blot analysis, different time points (30 minutes, 4 hours and 24 hours) were applied to cover the expression kinetics of the protein. Osteogenic induced BM MSC (day 3 after induction) served as positive control for the ERK1/2 activation (Figure 3) since the activation of ERK1/2 is required to induce the expression of matrix producing genes, such as collagens, *bone morphogenetic proteins* (BMPs) or *transforming growth factors* (TGFs). Surprisingly, while the non-phosphorylated ERK1/2 was expressed under all conditions in CB MSC, a DLK-1-specific phosphorylation of ERK1/2 was not detected. However, it has to be noted that a reduced adipogenic differentiation was observed in the CB MSC overexpressing the full-length *DLK-1* [3].

### 3.3. DLK-1 and Its Possible Role in Haematopoiesis-Supporting Capacity

Besides the inhibitory role on adipogenic differentiation of stromal cell populations, DLK-1 is also an important regulator of haematopoeisis [16, 17]. In 2010, data of Chou and Lodish clearly demonstrated that a DLK-1-positive subpopulation of murine fetal liver cells had a high impact on haematopoietic support [15]. Stromal cells, like USSC, CB MSC or BM MSC, are suitable feeder layers for CD34^+^ cell co-culture* ex vivo*. As published in 2005 expansion of cord blood-derived CD34^+^-cells on likewise CB-derived stromal cells leads to higher expansion rates compared to BM MSC [1] while *in vivo* cotransplantation of USSC into immuno-deficient mice resulted in improved engraftment [22]. 

**Figure 4:**
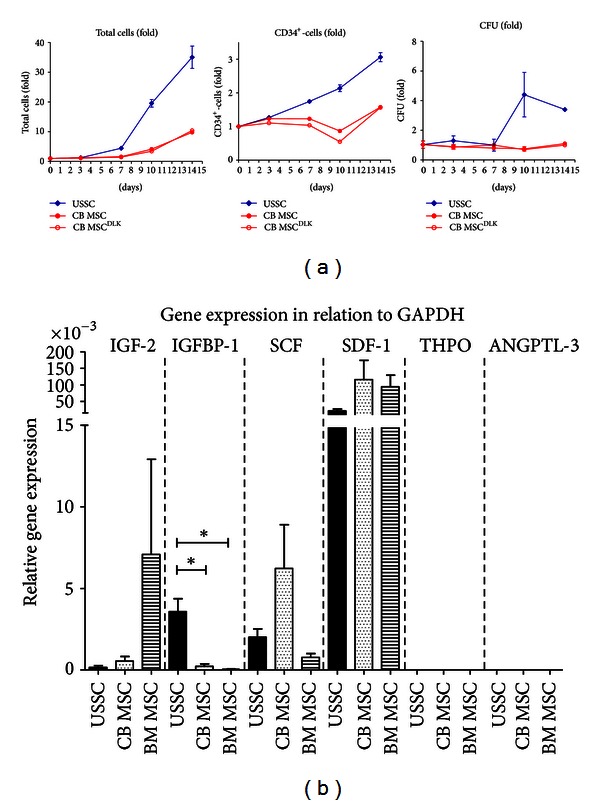
Haematopoiesis-supporting capacity of USSC and CB MSC. (a) Representative co-culture of CD34^+^-cells on feeders of USSC, CB MSC, and CB MSC overexpressing DLK-1 (CB MSC^DLK^) in multiple independent wells (*n* = 6). With regards to total cell count, expansion of CD34^+^-cells and colony-forming units (CFU), co-culture on a USSC-feeder resulted in higher expansion rates than on CB MSC-feeder. Within 14 days, total cell count increased significantly stronger on USSC-feeder (by factor 35.05 ± 3.75) than on CB MSC-feeder (factor 10.09 ± 0.37; *P* < 0.0001). Expansion of total CD34^+^-cells also resulted in significantly higher expansion on USSC (3.06 ± 0.08-fold) than on CB MSC (1.57 ± 0.02 fold; *P* < 0.0001). For colony-forming units, cells cultured on USSC-feeder showed a higher expansion rate on day 10 and 14 (up to 4.39 ± 1.50-fold versus 1.06 ± 0.03-fold; *P* = 0.1562). Overexpression of DLK-1 in the CB MSC line did not have an influence on expansion rates, as the results for CB MSC and CB MSC^DLK^ were approximately identical. (b) Expression levels of haematopoietic cytokines (*IGF-2*, *IGFBP-1*, *SCF*, *SDF-1*, *THPO*, *ANGPTL-3*) in USSC, CB MSC, and BM MSC were analyzed by real time PCR. The expression of insulin-like growth factor binding protein (*IGFBP-1*) was highest in USSC (*n* = 3), while stromal-derived factor 1 (*SDF-1*) expression was higher in CB MSC (*n* = 3) and BM MSC (*n* = 3) compared to USSC. Thrombopoietin (*THPO*) and angiopoietin-like 3 (*ANGPTL-3*) were not detectable.

However, with regard to the discrimination into USSC and CB MSC, here we demonstrate for the first time that only USSC possess an increased expansion capacity (Figure 4(a)) while CB MSC lead to expansion rates comparable to those achieved on BM MSC-feeder (data not shown). To evaluate the impact of DLK-1, co-culture experiments were also performed with CB MSC overexpressing DLK-1 (CB MSC^DLK-1^). In detail, application of USSC-feeders resulted in higher maximum fold-expansion rates than CB MSC-feeders with regard to total cell count (35.05 ± 3.75 versus 9.78 ± 0.52, *n* = 6 each, day 14) as well as CD34^+^ cell count (3.06 ± 0.08 versus 1.58 ± 0.037, *n* = 3 each, day 14). Additionally, an efficient expansion of colony-forming units (CFU) was observed only on USSC-feeder (4.39 ± 1.50, *n* = 2, day 10). 

Surprisingly, no differences in regard to expansion of total cells, CD34+ cells and CFU were detectable between the native CB MSC and the CB MSC^DLK-1^.

The DLK-1^+^ fetal liver subpopulation as described by Chou and Lodish expressed various haematopoietic growth factors, such as insulin-like growth factor-2 (IGF-2), insulin-like growth factor binding protein-1 (IGFBP-1), thrombopoietin (THPO), angiopoetin like-3 (ANGPTL-3), stem cell factor (SCF), and stromal-derived factor-1 (SDF-1). Therefore, the expression of these cytokines was evaluated in USSC, CB MSC, and BM MSC by quantitative real time PCR. The relative expression of *IGF-2*, *SCF*, and *SDF-1* was higher in CB MSC, while USSC expressed significantly higher levels of *IGFBP-1* (3.57∗10^−3^ ± 0.80∗10^−3^, *n* = 3) than CB MSC (0.21∗10^−3^ ± 0.16∗10^−3^, *n* = 3; *P* = 0.0147) and BM MSC (0.04∗10^−3^ ± 0.02∗10^−3^, *n* = 3; *P* = 0.0117). *THPO *and* ANGPTL-3* were not expressed in any of the cell types tested (Figure 4(b)).

**Figure 5:**
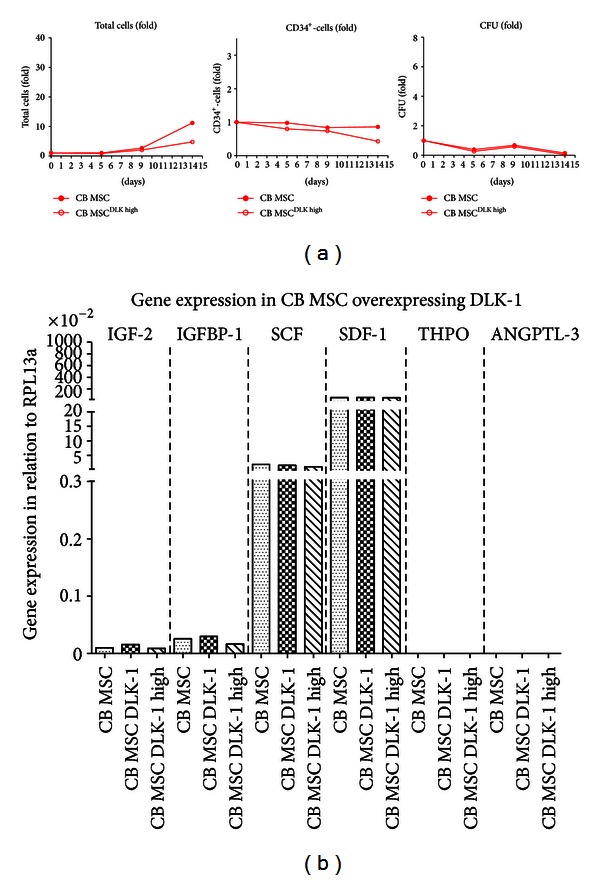
Effect of DLK-1-overexpression on the haematopoiesis-supporting capacity of CB MSC. (a) Exemplarily co-culture of CD34^+^-cells on feeders of CB MSC and FACS-sorted CB MSC strongly overexpressing DLK-1 (CB MSC^DLK  high^) in multiple independent wells (*n* = 4). Within 14 days, overexpression of DLK-1 did not have a beneficial effect on expansion rates of total cells, CD34^+^-cells or CFU. In accordance to former results for native CB MSC, only a weak expansion of total cells was observed, while amounts of total CD34^+^-cells as well as CFU slightly declined over time. (b) Expression levels of haematopoietic cytokines (*IGF-2*, *IGFBP-1*, *SCF*, *SDF-1*, *THPO*, *ANGPTL-3*) were analyzed by real time PCR exemplarily for a CB MSC line in native form, after induced overexpression of DLK-1 (CB MSC^DLK^) as well as after further FACS-sorting for cells with high DLK-1 expression (CB MSC^DLK  high^), respectively. No significant differences in expression levels could be detected and results were in accordance with former data, as presented in Figure 4(b).

Furthermore, even when exemplarily enriching the overexpressing CB MSC for the strongest presence of DLK-1 (CB MSC^DLK-1  high^) by fluorescent activated cell sorting (FACS), no significant differences were observed (Figure 5(a)). In accordance with these findings, DLK-1 overexpression also did not have an impact on the expression levels of the analyzed haematopoietic cytokines (Figure 5(b)).

## 4. Discussion

CB MSC and USSC are two distinct stromal cell populations in cord blood as confirmed by the different expression profiles in regard to *DLK-1*, *HOX* genes and CD146 [4, 19]. 

To further analyze the differences between these cell lines, previous experiments of our group were reanalyzed. These data verify a higher haematopoiesis-supporting capacity in USSC than in CB MSC or BM MSC as well as differences in the expression of various genes influencing haematopoiesis. Unexpectedly the expression of SCF, which is regarded as one of the most potent haematopoietic cytokines [23, 24], was higher in CB MSC than in USSC or BM MSC. Since expansion rates by co-culture on feeder layers of CB MSC were comparable to those on BM MSC and lower than on USSC, a beneficial effect by SCF possibly is counteracted by another factor. Here, as a potential antagonist, SDF-1 has to be discussed. Although mostly known as chemoattractant for haematopoietic stem cells [25], it can also induce quiescence and therefore reduce proliferation in HSC [26]. In summary, these results confirm that DLK-1-positive USSC have an increased haematopoiesis-supporting capacity. Whether this is directly related to DLK-1 as it was described by Moore et al. [16] or to other factors (such as IGFBP-1 or SCF/SDF-1; either secreted or presented via cell-cell contacts), still has to be clarified. In regard to the fact that USSC lack the secretion of the soluble form and overexpression in CB MSC did not show an effect on the expansion of CD34^+^-cells, a functional influence of DLK-1 could not be verified.

Moreover, the lack of extracellular expression and secretion of DLK-1 by USSC questions what is known about its function in adipogenesis in humans. In the murine model (3T3-L1 cells), the soluble part of DLK-1 (Pref1) is the functional adipogenic inhibitory protein [20] and the human form FA1 is highly abundant in serum [27]. Therefore, it has been postulated that native cord blood-derived stromal cells may homogenously lack adipogenic differentiation capacity due to inhibition by DLK-1 present in the plasma and that the adipogenic differentiation potential is artificially evoked due to dexamethasone in the culture media. However, since all cell lines are generated by the same protocol, this would not explain the occurrence of both cell types which has also been confirmed by other groups [5]. Additionally, both subpopulations can also be generated from the same cord blood by application of cloning cylinders. 

It has to be noted that DLK-1 belongs to the Delta-Notch family [28], where regulation of transmembrane proteins by endosomal trafficking is one of the key signal mechanisms. This has been studied in detail (for review see Le Borgne et al. 2005) [29], but whether the intracellular localization of DLK-1 observed in this study can be ascribed to this mechanism remains to be analyzed. Another possible explanation for the lack of extracellular expression of DLK-1 might be an alternative splice variant of the DLK-1 protein since 4 alternatively spliced DLK-1/ Pref1 variants have already been described in murine 3T3-L1 cells, with only the 50 kDa form being functional [21]. So far studies of human DLK-1 cleavage revealed only one soluble form with different molecular weights, due to different stages of glycosylation [18, 30].

However, the molecular changes induced by DLK-1/Pref1 in human MSC analyzed here are not consistent with the mechanisms described for several murine model systems (namely PPAR*γ*2, CEBP*α* expression and ERK1/2 phosphorylation) and relativize the hypothesis for DLK-1 to be the main regulator of adipogenesis in human cord blood-derived stromal cells.

Interestingly, IGFBP-1, which is higher expressed in USSC, is also known to be involved in the regulation of adipogenesis: Briefly, IGFs were shown to be equipotent to insulin in regulating adipogenic differentiation [31, 32] and the binding of IGF1 and IGBFPs leads to stable complexes that reduce the availability of free IGF1. As described by Nueda et al. [33], this complex can interact with the protease-target region of DLK-1 and further minimize the adipogenic potential of a cell by activation of ERK1/2.

## 5. Conclusion

The data presented here allow further characterization of cord blood-derived stromal cells, USSC and CB MSC, based on a total of 90 established and characterized cell lines (as of today, a total of 394 cell lines has been generated out of 1009 cord blood units).

According to their distinct properties, most prominently the presence of longer telomeres associated with a higher proliferative potential and a HOX-negative expression profile, USSC are regarded as being of a biologically younger state. Another functional difference is the lacking adipogenic differentiation potential of USSC, which was discussed as being possibly related to the expression of DLK-1 [3, 4]. 

In addition to these features, the data provided here demonstrate that USSC also can be distinguished from CB MSC and BM MSC by their higher haematopoiesis-supporting capacity in co-culture experiments. In contrast, CB MSC show properties similar to stromal cells found in the adult bone marrow, for example, lack of DLK-1 expression in association with a high adipogenic capacity and *HOX*-gene expression (although they still possess a higher proliferative potential and have longer telomeres than BM MSC). 

These features substantiate the assumption that among neonatal stromal cells, CB MSC are closer related to bone marrow stromal cells than USSC. As an amendment to our definition of USSC, DLK-1 remains a useful marker to distinguish USSC from CB MSC but since the data presented in this study confirm the absence of extracellular DLK-1 expression and the lack of protein release into the supernatant, its specific role remains unclear. Accordingly, an adipogenic inhibitory function based on DLK-1 alone in USSC is unlikely, although a functional interaction with other factors, such as IGFBP1, needs to be analyzed. The same accounts for the effect of DLK-1 on haematopoiesis, since overexpression of DLK-1 in CB MSC did not result in an increased supportive capacity comparable to USSC. 

Therefore, further analyses of USSC are required to test whether they have yet unknown qualities beyond those of “classic” MSC and if this is related to a different origin of USSC and CB MSC, respectively. Additionally, whether regulations during adipogenesis in human and mice are subject to the same molecular mechanisms, especially in regard to the importance of DLK-1, has to be clarified.

## Figures and Tables

**Table 1:** Primer applied in this study.

Gene		Primer Sequence: 5′-3′	Product (bp)	Annealing temperature
Angiopoetin-like 3 (ANGPTL-3)	NM_014495.2	Forward: TGGGAGGCTTGATGGAGAAT Reverse: GTAGCGTATAGTTGGTTTCG	164 bp	60°C
CCAAT enhancer binding protein alpha (CEBP*α*)	NM_004364.3	Forward: GAGTCACACCAGAAAGCTAG Reverse: GATGGACTGATCGTGCTTC	184 bp	60°C
*Delta like-1 homologue (DLK-1) *	NM_003836.5	Forward: AGCACCTATGGGGCTGAAT Reverse: GTCACGCACTGGTCACAAAG	119 bp	60°C
*Glyceraldehyde-3-phosphate dehydrogenase (GAPDH) *	NM_002046.4	Forward: GAGTCAACGGATTTGGTCGT Reverse: TTGATTTTGGAGGGATCTCG	238 bp	60°C
*Insulin-like growth factor 2* (IGF-2)	NM_000612.4, NM_001007139.4, NM_001127598.1	Forward: ATTGCTGCTTACCGCCCCA Reverse: CACTCCTCAACGATGCCACG	143 bp	60°C
*Insulin-like growth factor-binding protein 1* (IGFBP-1)	NM_000596.2	Forward: CAAAAAATGGAAGGAGCCC Reverse: GATGTCTCACACTGTCTGC	153 bp	60°C
*Peroxisome proliferators activator gamma 2 (PPAR*γ*2) *	NM_015869.4	Forward: TCCATGCTGTTATGGGTGAA Reverse: TCAAAGGAGTGGGAGTGGTC	193 bp	60°C
*Ribosomal protein L13a (RPL13a) *	NM_001270491.1, NM_012423.3	Forward: GAGGTATGCTGCCCCACAAA Reverse: TTCAGACGCACGACCTTGAG	136 bp	60°C
*Stem cell factor * (SCF)	NM_003994.5, NM_000899.4	Forward: GGATAAGCGAGATGGTAGTA Reverse: CTTCAGGAGTAAAGAGCC	210 bp	55°C
*Stromal-derived factor 1 *(SDF-1)	NM_199168.3, NM_000609.5, NM_001033886.2, NM_001178134.1	Forward: CTCAACACTCCAAACTGTGC Reverse: GCTTTCTCCAGGTACTCC	110 bp	60°C
Thrombopoietin (THPO)	NM_000460.2, NM_001177597.1, NM_001177598.1	Forward: TCACCCTTTGCCTACACC Reverse: GTCACTGCTCCCAGAATGT	108 bp	60°C

**Table 2:** Antibodies utilized in this study.

Primary Antibody	Clone	Company
DLK-1	PF299-1	Enzo life Science; Farmingdale, NY, USA
ERK1/2	16/ERK	BD Transduction Laboratories
p-ERK1/2	E4	Santa Cruz; Dallas, TX, USA
*β*-Actin	AC-74	Sigma-Aldrich
CD34-PE	8G12	BD Bioscience
CD45-FITC	2D1	BD Bioscience
Secondary Antibody		
Immunofluorescence: Rhodamine Red-X-conjugated goat antimouse IgG		Dianova; Hamburg, Germany
Flow cytometry: FITC-conjugated goat antimouse		Jackson ImmunoResearch Inc.; West grove, PA, USA
Western blot: HRP-conjugated goat antimouse IgG	sc-2005	Santa Cruz
